# Maize leaf PPDK regulatory protein isoform-2 is specific to bundle sheath chloroplasts and paradoxically lacks a Pi-dependent PPDK activation activity

**DOI:** 10.1093/jxb/erx471

**Published:** 2017-12-21

**Authors:** Chris J Chastain, Lisa M Baird, Mitchell T Walker, Charles C Bergman, Gulnara T Novbatova, Candida S Mamani-Quispe, Jim N Burnell

**Affiliations:** 1 Department of Biosciences, Minnesota State University-Moorhead, USA; 2 Department of Biology, University of San Diego, San Diego, CA, USA; 3 Department of Molecular and Cell Biology, James Cook University, Australia

**Keywords:** C_4_ pathway, C_4_ photosynthesis, PPDK regulatory protein, PPDK, *Zea mays*, *Zm*PDRP1, *Zm*PDRP2

## Abstract

In C_4_ plants, the pyruvate phosphate dikinase regulatory protein (PDRP) regulates the C_4_ pathway enzyme pyruvate phosphate dikinase (PPDK) in response to changes in incident light intensity. In maize (*Zea mays*) leaves, two distinct isoforms of PDRP are expressed, *Zm*PDRP1 and *Zm*PDRP2. The properties and C_4_ function of the *Zm*PDRP1 isoform are well understood. However, the PDRP2 isoform has only recently been identified and its properties and function(s) in maize leaves are unknown. We therefore initiated an investigation into the maize PDRP2 isoform by performing a side by side comparison of its enzyme properties and cell-specific distribution with PDRP1. In terms of enzyme functionality, PDRP2 was found to possess the same protein kinase-specific activity as PDRP1. However, the PDRP2 isoform was found to lack the phosphotransferase activity of the bifunctional PDRP1 isoform except when PDRP2 in the assays is elevated 5- to 10-fold. A primarily immuno-based approach was used to show that PDRP1 is strictly expressed in mesophyll cells and PDRP2 is strictly expressed in bundle sheath strand cells (BSCs). Additionally, using *in situ* immunolocalization, we establish a regulatory target for PDRP2 by showing a significant presence of C_4_ PPDK in BSC chloroplasts. However, a metabolic role for PPDK in this compartment is obscure, assuming PPDK accumulating in this compartment would be irreversibly inactivated each dark cycle by a monofunctional PDRP2.

## Teaser

In the maize C_4_ pathway, PDRP1 is known to regulate C_4_ PPDK activity in mesophyll chloroplasts. A second PDRP isoform, PDRP2, is shown to be the apparent regulator of C_4_ PPDK in bundle sheath chloroplasts.

## Introduction

**Fig. 1.**
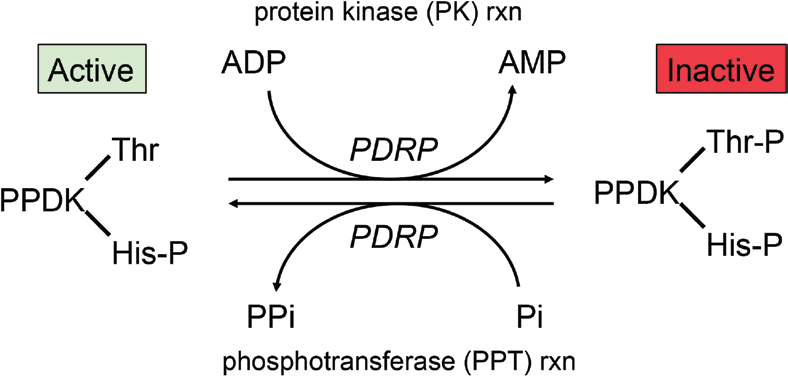
Reversible phosphorylation of PPDK by PDRP. Inactivation of PPDK by PDRP proceeds by phosphorylation of an active site threonine residue (Thr456 in maize). Only the E-His-P catalytic intermediate enzyme form, as indicated by the His-P residue (His458 in maize), is amenable to PDRP phosphorylation. Reactivation of PPDK is catalyzed by Pi-dependent dephosphorylation of this same target residue by PDRP’s phosphotransferase activity. (This figure is available in colour at *JXB* online.)

**Fig. 2.**
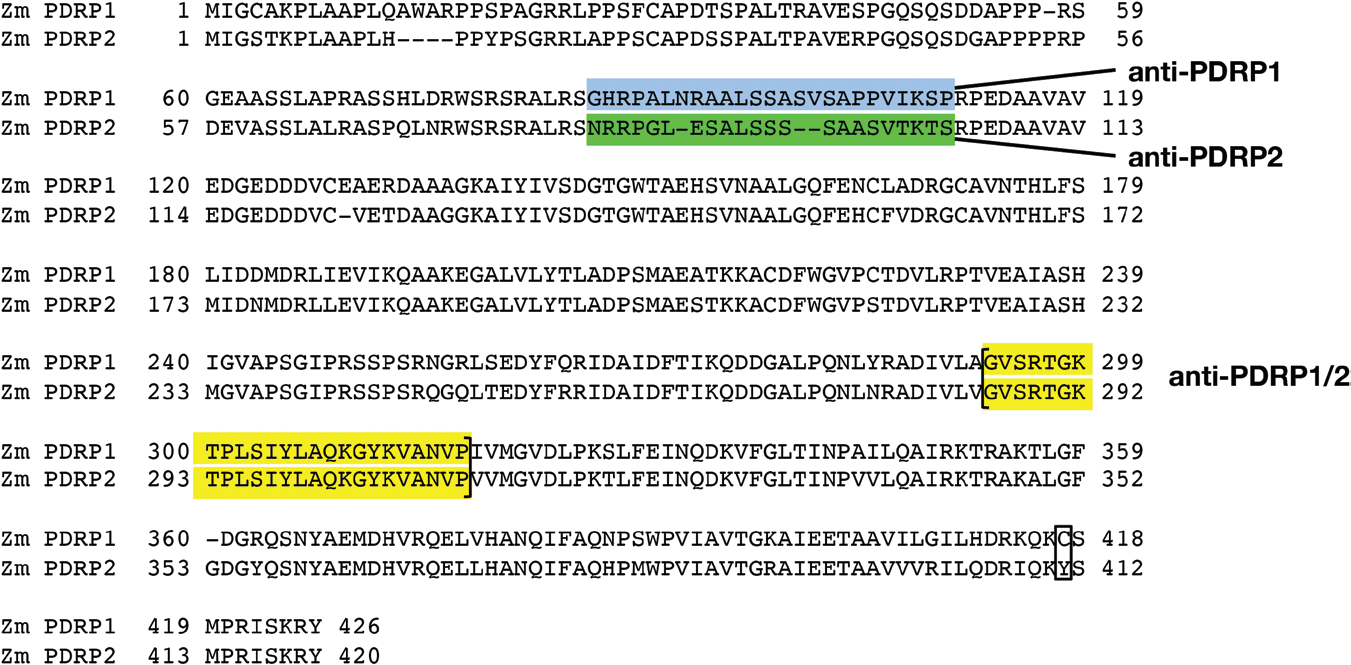
ClustalW alignment of *Zm*PDRP1 versus *Zm*PDRP2. Highlighted regions are the sequences of peptides used as epitopes for generating the PDRP antibodies used in this study. A cysteine residue demonstrated to form an intramolecular disulfide bond in PDRP1 (Cys417) (Jiang *et al.*, 2016) and the corresponding PDRP2 residue, Tyr411, are indicated in a box at the C-terminal portion of the sequences. (This figure is available in colour at *JXB* online.)

In the C_4_ metabolic pathway, the pyruvate, phosphate dikinase (PPDK) regulatory protein (PDRP) regulates PPDK activity according to the level of incident light (Chastain, 2011). Mechanistically, PDRP regulates PPDK in a strict on/off fashion via its ability to phosphorylate reversibly a threonine residue (Thr456 in maize) within the PPDK active site (Fig. 1). When this residue is phosphorylated, PPDK is inactive, and vice versa. In C_4_ plants, PDRP is known to be co-localized with C_4_ PPDK in the stromal compartment of mesophyll cell (MC) chloroplasts. In maize, PDRP was assumed to be encoded by a single gene, *ZmPDRP1* (GRMZM2G131286), corresponding to the first reported PDRP gene cloned in 2006 (Burnell and Chastain, 2006). However, with the release of the higher resolution version-2 maize B73 genome, a second PDRP gene, *ZmPDRP2* (GRMZM2G004880), was revealed, along with transcript evidence of its expression in maize leaves (Andorf *et al.*, 2016). The two isoforms of the protein are highly similar in amino acid sequence (83% identical, 7% similar, Fig. 2). Both genes are predicted to encode N-terminal chloroplast transit peptides by ChloroP (Emanuelsson *et al.*, 1999). Although transcripts from both genes have been shown to be primarily expressed in green tissues, three studies that characterized the transcriptomes of isolated maize leaf bundle sheath cells (BSCs) and MCs (Chang *et al.*, 2012; Tausta *et al.*, 2014; Denton *et al.*, 2017) revealed that PDRP1 transcript was specific to MCs and PDRP2 transcript was specific to BSCs, with minor overlap attributed to cross-contamination of isolated cell types. The finding that a separate chloroplast-targeted PDRP isoform was expressed in BSCs was somewhat unexpected, owing to the fact that PPDK carries out its function in the C_4_ pathway in MC chloroplasts. Furthermore, C_4_ PPDK, as for the other MC C_4_ enzymes, was initially understood to be present in very low amounts in BSC chloroplasts (Kanai and Edwards, 1999). Moreover, according to the current models of dual-cell C_4_ photosynthesis, PPDK activity in this compartment would likely impair C_4_ cycle function by perturbing the recycling of pyruvate [or its three-carbon organic acid equivalent (Arrivault *et al.*, 2017)] back to the mesophyll for regeneration into the primary CO_2_ acceptor phosphoenolpyruvate (PEP) by C_4_ PPDK (von Caemmerer and Furbank, 1999; Bellasio and Griffiths, 2014; Wang *et al.*, 2014). Nonetheless, the finding that the transcript of a second PDRP gene, PDRP2, is specifically expressed in BS chlorenchyma suggests a presence of C_4_ PPDK in BSC chloroplasts substantial enough to require regulation. Indeed, both the aforementioned RNA sequencing (RNA-seq) studies found the abundance of C_4_ PPDK transcript in BSCs to be quite high at 22% (Chang *et al.*, 2012) and 35% (Tausta *et al.*, 2014), respectively, of the high transcript level of MCs. Ueno (1998), using a quantitative *in situ* immunolocalization method, also found higher levels of PPDK protein in maize BSC chloroplasts (BSC/MC PPDK ratio of 0.35). Within this study, only *Sorghum*, with a BSC/MC PPDK ratio of 0.15, had a similarly high PPDK level. None of the other seven C_4_ grasses examined were found to have as high a level of BSC plastid PPDK (BSC/MC ratios of 0.03–0.10). Additionally, because of the strict expression of the PDRP isoform transcripts in alternate chlorenchyma cell types, we conjectured that PDRP2 likely fulfills a PPDK-regulatory role that differs from the regulatory role PDRP1 fulfills in its regulation of C_4_ PPDK in MC chloroplasts. In order to validate such conjecture, the present investigation was initiated with the objective of establishing that expression of the PDRP polypeptides corresponds to the same cell-specific expression found for their transcripts: PDRP1 transcript in MCs and PDRP2 transcript in BSCs. A second objective was to demonstrate that the PDRP2 isoform was enzymatically competent in conferring phospho-regulation of PPDK. Finally, a third line of evidence needed was an unequivocal demonstration that C_4_ PPDK, the putative regulatory target for PDRP2, was present at substantial levels in BSC chloroplasts. Using a largely immunological approach, we provide evidence in this report supporting all three objectives. Taken together, these findings suggest a functional role for PDRP2 in C_4_ photosynthesis which is distinctly different from the C_4_ role which PDRP1 fulfills in MCs.

## Materials and methods

### Plant material

*Zea mays* seeds were obtained from Peterson Farms Seed, Fargo, ND, USA (hybrid 57H87). With the exception of field-grown plants used for the reverse transcription–quantitative PCR (RTqPCR) experiments, all leaf material originated from plants that were greenhouse germinated and grown. Seeds were planted and germinated in 2 gallon plastic nursery pots in a potting-soil mix (Metro-Mix 900, Sun Gro Horticulture, Inc., Canada) supplemented with Osmocote Plus™ 15-8-11 controlled-release fertilizer. During growth, plants were alternately watered with a 15-5-15 water-soluble fertilizer. Plants were grown under supplemental lighting [450 µmol photon m^−2^ s^−1^ photosynthetically active radiation (PAR), 14/10 h day/night cycle] and a day/night temperature of 28 °C/24 °C. Field-grown *Z. mays* plants used for qPCR analysis of transcript were cultivated during the summer on a plot adjacent to the MSUM campus using the same watering/fertilizing regimen as above. Leaf material used for RNA isolations was harvested as 1.0 cm disks taken from the mid-center portion of fully expanded leaves at mid-day/mid-summer and immediately flash-frozen in liquid nitrogen for subsequent storage at –80 °C before use.

### Isolation of leaf bundle sheath strands and mesophyll cell protoplasts

For isolation of MC protoplasts and BSC strands, the center portion of fully expanded leaves from 6- to 7-week-old plants harvested at mid-day were used. An enzyme digestion-based protocol was used to obtain MC protoplasts from fully expanded maize leaves (Chang *et al.*, 2012). The purity of isolated protoplasts was confirmed by examining preparations with a light microscope and later by immunoblot analysis of extracted proteins (see Supplementary Fig. S2 at *JXB* online). Immediately upon isolation, aliquots of protoplasts were combined with an equal volume of Trizol™, vortexed for 30 s for cell lysis, and then stored at –20 °C until used. For isolation of BSC strands, a mechanical separation-based protocol was used (Agostino *et al.*, 1989) with the following modifications. Freshly harvested and de-ribbed leaves (~10 g) were first made fully turgid by immersion in distilled H_2_O (~22 °C) for 10 min, daubed with paper towels to remove excess H_2_O, and then sliced transversely into 0.5 cm pieces and placed into a Waring blender along with 120 ml of ice-cold extraction medium (0.35 M sorbitol, 25 mM HEPES-KOH, pH 7.5, 2 mM MgCl_2_, 2 mM KPi, 2 mM EDTA, and 2 mM sodium iso-ascorbate). The leaves were blended for 10 s at 60% of line voltage and then for an additional 2–3 bursts of 10 s each at 40% of line voltage. BSC strands were separated from mesophyll and epidermis tissue by filtering the green slurry successively through 1 mm and 650 µm nylon mesh nets and finally through two layers of pre-wetted miracloth. The BSC strands retained on the miracloth were washed twice with ice-cold resuspension medium (0.3 M sorbitol, 20 mM HEPES-KOH pH 7.5, 0.5 mM KPi, 10 mM KCl, 2 mM EDTA). The washed BSC strands were collected from the miracloth using a micro-spatula and immersed in 50 ml of ice-cold resuspension medium. After 5 min, the BSC strands settled to the bottom, with residual epidermal cells remaining in the medium above the strands, which was removed by aspiration. The BSC strands were then re-collected on pre-wetted miracloth and washed again with resuspension medium as before. The washed strands were collected as above and deposited in batches into 1.5 ml microtubes and immediately frozen at –20 °C for later use. Prior to freezing, each BSC strand preparation was inspected with a light microscope to confirm that the strands were free of MCs and epidermal tissue, and later by immunoblot analysis of extracted proteins (Supplementary Fig. S2).

### Extraction of proteins from intact leaf tissue, mesophyll protoplasts, and bundle sheath strands

Proteins were extracted from cells and leaf tissue with Trizol™, a mono-phase solution of phenol and guanidine thiocyanate, using the manufacturer’s protocol (https://www.mrcgene.com/rna-isolation/tri-reagent) with the following variations. For extraction of protein from intact leaf material, a mortar and pestle were used to produce a Trizol–leaf homogenate. Frozen lots of MC protoplasts and BSC strands were homogenized in Trizol using a glass homogenizer. Protein precipitates isolated by this procedure were solubilized in 1% sarkosyl and directly combined with SDS–PAGE sample buffer for subsequent denaturing gel electrophoresis. Solubilized protein was quantified by a modified Coomassie dye binding method (Coomassie Plus™, ThermoFisher Scientific) with crystalline BSA as standard.

### Immunoblot analysis

SDS–PAGE and immunoblotting procedures were performed as described previously and include the following modifications (Chastain *et al.*, 2008). For SDS–PAGE, Bolt™ Bis-Tris pre-cast acrylamide gels (10% or 12%) were used. The primary antibodies used in immunoblot procedures were: an affinity-purified (rabbit) polyclonal antibody raised against maize recombinant C_4_ PPDK (Chastain *et al.*, 2008); an affinity-purified (rabbit) polyclonal antibody raised against a phosphopeptide corresponding to the threonine phosphorylation domain of maize C_4_ PPDK (Chastain *et al.*, 2006); an affinity-purified (rabbit) polyclonal anti-PDRP1 antibody, generated against *Zm*PDRP1 residues 86–110 (GHRPALNRAALSSASVSAPPVIKSP; see Fig. 2); an affinity-purified (rabbit) polyclonal anti-PDRP2 antibody, generated against *Zm*PDRP2 residues 83–104 (NRRPGLESALSSSSAASVTKTS; see Fig. 2]; and anti-PDRP1/2 antibody, an affinity-purified (rabbit) polyclonal antibody generated against a peptide common to both PDRP isoforms, *Zm*PDRP1 residues 293–317 and *Zm*PDRP2 residues 286–310 (GVSRTGKTPLSIYLAQKGYKVANVP; see Fig. 2). For use as a lane loading control on immunoblots, an affinity-purified polyclonal (rabbit) antibody against an Arabidopsis actin peptide was obtained from Agrisera AB, Sweden (cat.# AS13 2640), as were affinity-purified polyclonal peptide antibodies against PEP carboxylase (PEPC; cat.# AS09 458) and Rubisco large subunit (LSU; cat.# AS03 037A). The latter two antibodies were used for testing cell type cross-contamination of MC and BSC isolations. A goat anti-rabbit alkaline phosphatase (AP) conjugate (Promega) was used as the secondary antibody in all immunoblot procedures. For signal detection, immunoblots were incubated with the chemiluminesence substrate CDP-Star™ and digitally scanned using a Li-Cor C-Digit blot scanner (Li-Cor Biosciences, Lincoln, NE, USA).

### Expression and purification of recombinant enzymes

Plasmids used for generating recombinant *Z. mays* C_4_ PPDK and PDRP1 have been described previously (Burnell and Chastain, 2006; Chastain *et al.*, 2011). A codon-optimized *Z. mays* PDRP2 sequence encoding amino acids 1–420 (GRMZM2G004880_P01) was synthesized by Aldevron LLC (Fargo, ND, USA) and spliced into the *Escherichia coli* expression vector pET 28a for subsequent expression of recombinant PDRP2 enzyme. Host *E. coli* BL21 DE3 cells were transformed with the respective plasmid and used for large-scale culture as described before (Chastain *et al.*, 2008, 2011). Subsequent extraction and Ni-NTA affinity purification of 6×His-tagged recombinant proteins were accomplished as previously described (Chastain *et al.*, 2011). Affinity-purified enzyme was precipitated in 70% ammonium sulfate, aliquoted into 1.5 ml microtubes, and pelleted by centrifugation at 14 000 *g*. Drained pellets were flushed and sealed in N_2_, then stored at –80 °C. Prior to use in assays, the recombinant PDRP enzymes were reconstituted in buffer consisting of 50 mM Bicine, pH 8.3, 5 mM MgSO_4_, 0.1 mM EDTA, 5 mM DTT, and 1 mg ml^−1^ blue dextran, while recombinant C_4_ PPDK was reconstituted in 50 mM Bicine, pH 8.3, 10 mM MgSO_4_, 5 mM DTT, and 5% glycerol. Prior to use in assays, resuspended enzymes were desalted using a Zeba™ spin-column (ThermoFisher Scientific, USA).

### PDRP enzyme assays

**Fig. 6.**
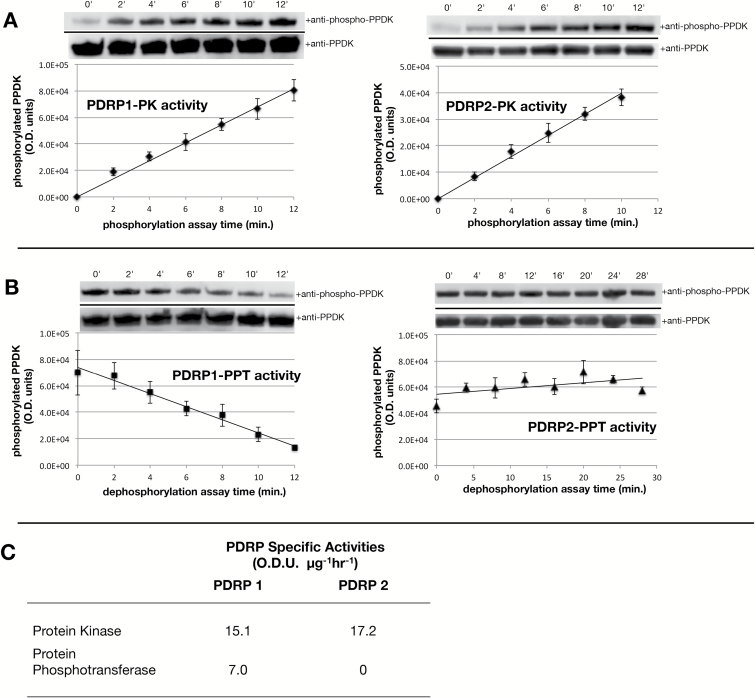
*In vitro* PDRP enzyme assays. Recombinant PDRP was assayed for protein kinase (PK; PPDK phosphorylation) and protein phosphotransferase (PPT; phospho-PPDK dephosphorylation) activity using an immuno-based assay as described in the Materials and methods. (A) PK assays. Above each plot is a representative immunoblot of time-point aliquots from a single assay as probed with phospho-PPDK antibody. Blots were stripped and reprobed with PPDK antibody as a lane load control. Below are plots showing PDRP-catalyzed PPDK phosphorylation as a linear increase in phospho-PPDK band signal intensity, as optical density units, versus time. Each plot is the mean of four separate PK assays. (B) PPT assays. Immunoblots and plots showing PDRP-catalyzed PPDK dephosphorylation as a decrease in phospho-PPDK band signal versus time. PPT assays of PDRP2 utilized an extended assay period (28 min versus 12 min). (C) Specific enzyme activities calculated from plots shown in (A) and (B). *n*=4, *R*^2^≥0.98;

Protein kinase (PK) and protein phosphotransferase (PPT) activities were assayed using a previously established immuno-based procedure with certain modifications (Chastain *et al.*, 2011; Tolentino, *et al.*, 2013). For assay of the PDRP–PK reaction, 190 µl reactions comprised of 70 µg of affinity-purified recombinant maize PPDK (fully activated, non-phospho-form), 50 mM Bicine-KOH, pH 8.3, 10 mM MgCl_2_, 5mM DTT, 1 mg ml^−1^ BSA, 1 mM ADP, 0.2 mM ATP, and 0.2 mM P1,P5-di(adenosine-5')-pentaphosphate (Ap5A) were first incubated at 30 °C for 10 min to ensure that recombinant PPDK monomers were fully tetramerized. The reactions were initiated by addition of 10 µl of 100 ng µl^−1^ PDRP. At 2 min intervals, 20 µl aliquots were removed and quenched in 20 µl of SDS–PAGE sample buffer. For assay of PDRP–PPT activity, PPDK-P substrate was first generated by a parallel PK reaction as described above, except that the PK reaction time was 25 min to ensure maximum phosphorylation of PPDK. The source of PDRP in the PPDK-P-generating reaction was either PDRP1 or PDRP2, depending on whether PDRP1 or PDRP2 was being assayed in the PPT reaction. PPDK-P substrate used in the PPT reaction was recovered from the PK reaction by rapid desalting through a Zeba desalting column (0.5 ml bed volume, 7K MWCO, pre-equilibrated with 50 mM Bicine-KOH, pH 8.3, 10 mM MgCl_2_, 5 mM DTT) to remove interfering first-step PK reaction metabolites. The desalted phospho-PPDK was then incubated for 5 min at 30 °C, and the PPT reaction initiated by simultaneous addition of KPi and PDRP to final concentrations of 2.5 mM and 10 ng ml^−1^, respectively. At 2 min intervals, 20 µl aliquots were removed and quenched in 20 µl of SDS–PAGE sample buffer. Rates of PDRP-catalyzed PPDK phosphorylation and dephosphorylation were obtained by quantitative immunoblot analysis of phospho-PPDK signal from 8 µl reaction aliquot bands of the terminated reaction (Fig. 6A, B). Only the linear portion of the reactions were used to compute specific activities. Image-Studio™ software (Li-Cor Bioscience) was used to quantitate band signals from the immunoblots digitally.

### Isolation of RNA and leaf tissue transcript quantitation using RTqPCR

Total RNA was isolated from field-grown maize leaf tissue using a Direct-zol™ RNA Kit (Zymo Research). Following isolation, total RNA was assayed for quantity and purity (*A*_260_/*A*_280_) using a Nanodrop UV-Vis spectrophotometer and examined for intactness using agarose gel electrophoresis (Supplementary Fig. S1). qPCR of reverse-transcribed C4PPDK, PDRP1, and PDRP2 mRNAs was accomplished by combining an aliquot of total RNA with a gene-specific hydrolysis probe mix (Taqman™ Gene Expression Assays, Applied Biosystems) and one-step, single-tube reverse transcription–qPCR reaction mix (1-Step Brilliant II™ QRT-PCR Master Mix, Agilent Technologies) for subsequent amplification of cDNA in a Stratagene 3000Mx optical thermocycler. Absolute transcript copy number per nanogram of total RNA was obtained from a standard curve of known amplicon copy concentration versus C_t_. A single 440 bp synthetic dsDNA containing the amplicon sequences of *Zm*C4PPDK, *Zm*PDRP1, and *Zm*PDRP2 was used as template for the standard curve qPCRs.

### *In situ* immunolocalization

Maize leaf samples from the mid-center portion of fully expanded leaves were fixed for 4 h at 4 °C in 2% (v/v) paraformaldehyde and 1.25% (v/v) glutaraldehyde in 50 mM PIPES buffer, pH 7.2. Samples were dehydrated in a graded ethanol series and flat-embedded in LR White-resin (Electron Microscopy Sciences, USA). Cross-sections were cut at 1 µm and dried from a drop of water onto Super Frost™ slides (ThermoFisher Scientific, USA). Slide-mounted tissue was blocked for 1 h in TBST [10 mM Tris, pH 7.2, 150 mM NaCl, 0.3% Tween-20 (v/v)] plus 1% BSA (w/v) and then incubated for 3 h in either pre-immune serum (1:100 dilution) in TBST+BSA or maize C_4_ PPDK antibody (1:50 dilution) in TBST+BSA. After washing with TBST+BSA, slides were treated for 1 h with goat anti-rabbit IgG gold antibody (Sigma-Aldrich, USA) diluted 1:100 in TBST+BSA. After washing, the sections were treated with silver enhancement reagent (Sigma-Aldrich, USA) for 10 min following the manufacturer’s instructions. Sections were washed well, counterstained for 2 min in 0.5% (w/v) aqueous Safranin O, and imaged with a Nikon A1R laser scanning confocal microscope. For Alexa staining, slide-mounted tissue was treated with PDRP2 antibody and washed as described above, then treated with goat anti-rabbit IgG Alexa 488 dye conjugate (ThermoFisher Scientific, USA) in TBST+BSA (1:500 dilution) for 30 min, rinsed with TBST+BSA, and imaged with the confocal microscope.

## Results

### Purity of MC protoplasts and BSC strand preparations

Virtually all methods used for isolating MC protoplasts and BSC strands from C_4_ leaves have the potential for cross-contamination (Edwards *et al.*, 2001; Chang *et al.*, 2012; Covshoff *et al.*, 2013; Denton *et al.*, 2017). We examined our preparations for cell type purity by probing immunoblots of protein extracted from our cell isolations with Rubisco LSU antibody, as a marker for BS cells, and PEPC antibody, as a marker protein for M cells. These results show that each isolation contained little, if any, signal for the respective cross-contamination marker protein (Supplementary Fig. S2). The normalizing of MC and BSC protein loads used for all immunoblots performed in this study is necessarily problematic. Due to the different complement of expressed proteins in the two differentiated cell types, no one housekeeping-type protein or pigment can serve as an absolute common denominator for normalizing protein loads. In light of this, an actin antibody was used in this study as primarily a relative indicator that lane protein loads between MC and BSC strand cell lanes were comparable in range. Typically, lanes of BSC strand protein had lower actin signal than MC and parent leaf lanes even though the total protein applied to each lane was the same (Supplementary Fig. S2).

### Validation of the PDRP antibodies used in this study

**Fig. 3.**
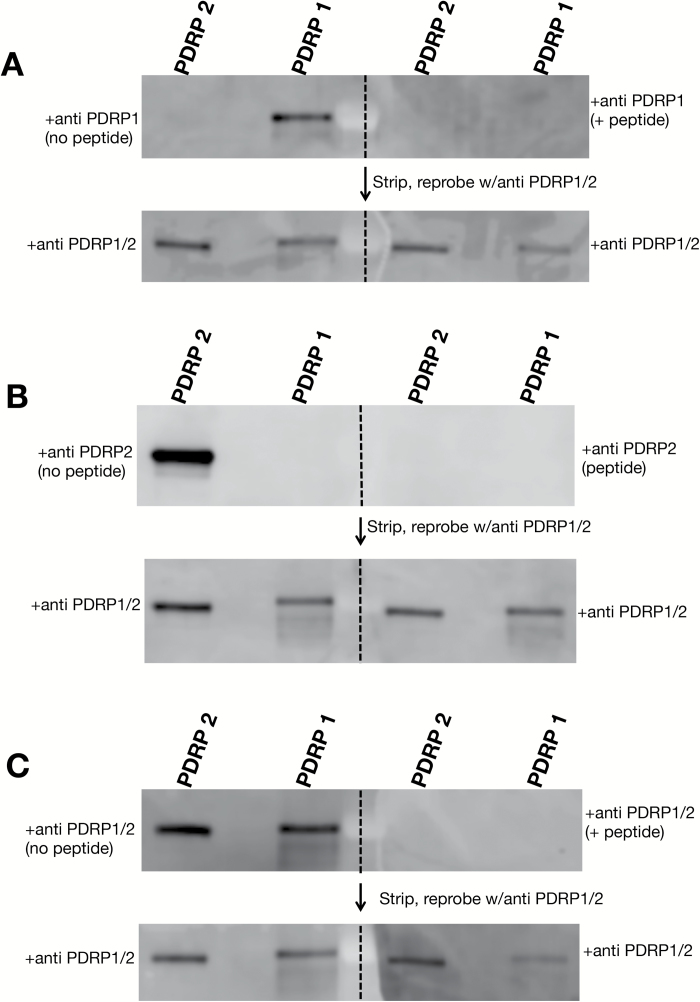
Specificity test of PDRP peptide antibodies used in this study. Immunoblots of recombinant PDRP1 and PDRP2 protein were hybridized with (A) anti-PDRP1, (B) anti-PDRP2, and (C) anti-PDRP1/2 antibodies in the absence (left side) or presence (right side) of the respective PDRP peptide used to generate each antibody. Dashed lines represent where the initial blot was separated into halves for hybridization as indicated. Following chemiluminescent detection, immunoblots were stripped of primary antibody and reprobed with PDRP1/2 antibody that targets a peptide epitope common to both PDRP1 and PDRP2 polypeptide as a control. Each lane contained 50 ng of purified, recombinant PDRP protein.

The high degree of amino acid similarity between the two PDRP isoforms presented a challenge in finding suitable sequences within the polypeptides that carried enough cross-variability to be used as isoform-specific epitopes for generating PDRP-specific antibodies yet be excluded from the variable N-terminal chloroplast transit region. We finally settled on a sequence of 25 amino acids just downstream from the predicted transit sequence as peptides for generating isoform-specific polyclonal antibodies in rabbits (Fig. 2). Antibodies generated from each peptide epitope were isolated from sera using a two-step affinity purification procedure. The purified antibodies were validated for specificity by probing replicate immunoblots of purified, recombinantly produced PDRP1 and PDRP2 protein with the respective PDRP isoform antibody, with and without inclusion of peptide antigen (Fig. 3A–C). When the blots were probed with anti-PDRP1 antibody, PDRP1 but not PDRP2 was detected (Fig. 3A). When PDRP1 antigen peptide was included in the hybridization buffer, the PDRP1 band signal was ablated. Similar specificity was shown by the PDRP2 antibody (Fig. 3B), where PDRP2 but not PDRP1 polypeptide was detected. This band could be ablated by inclusion of anti-PDRP2 peptide antigen with the antibody. A third PDRP peptide antibody used in this study, designated as anti-PDRP1/2 antibody, was generated using an amino acid sequence common to both isoforms (Fig. 2). This antibody detected both PDRP1 and PDRP2 polypeptides with no signal evident when the PDRP1/2 peptide antigen was included in the antibody hybridization (Fig. 3C). The PDRP1/2 antibody was also used as a control antibody to verify the presence of PDRP1 or PDRP2 protein on those test blots where these bands are absent. This was accomplished by stripping previously probed blots of primary antibody and reprobing with PDRP1/2 antibody alone (Fig. 3A–C).

### Transcript abundance of PDRP1 and PDRP2 genes in intact leaves

**Fig. 4.**
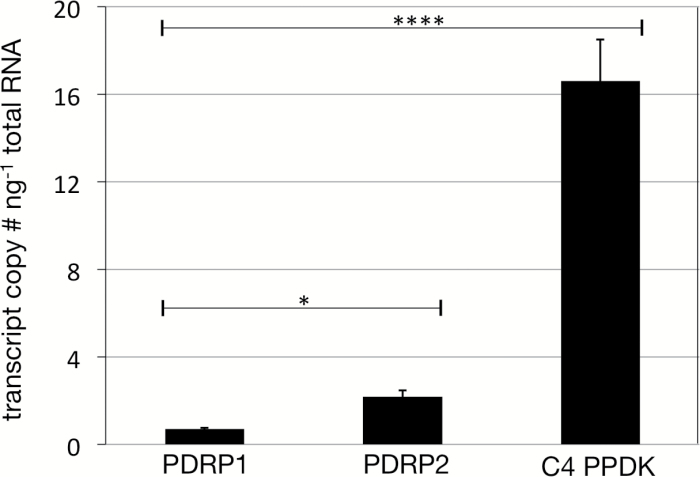
Absolute RTqPCR measurement of PDRP1, PDRP2, and C_4_ PPDK transcript levels in mature, field-grown maize leaves. A hydrolysis probe-based method was utilized to obtain gene-specific transcript abundance in isolated maize leaf RNA. Error bars indicate the SD of the means, *n*=6, *P<0.05; ****P<0.0001.

Previous RNA-seq studies using RNA isolated from intact leaves show wide variation between studies in the amount and proportions of PDRP1 and PDRP2 transcript (Wang *et al.*, 2009; Li *et al.*, 2010; Davidson *et al.*, 2011; Chettoor *et al.*, 2014; Tausta *et al.*, 2014). This most likely can be attributed to variations in leaf age, growth conditions, and even the portion of the leaf used for RNA isolation. What is clear from these reports, however, is that the transcript level for both PDRP genes is consistently in the lower range of all expressed genes in the leaf [e.g. 50–1000 fragments per kilobase of transcript per million mapped reads (FPKM)]. In terms of comparative abundance, these studies show that the PDRP1 transcript level is typically several fold higher than that of the PDRP2 transcript. In a study of transcriptomes from separated BSCs and MCs that included RNA-seq data for RNA isolated from intact leaves (Tausta *et al.*, 2014), the PDRP1 transcript level was found to be 2-fold higher than that of the PDRP2 transcript in separated cells but 5-fold higher than that in intact parent leaves. Because of the wide variation among these previous reports, we sought to gain more precise estimates of the PDRP transcript level in RNA isolated from mature, field-grown leaves using a hydrolysis probe-based qPCR method. An assay of the high-copy C_4_ PPDK transcript was also included to enable a relative comparison of PDRP transcript level. The results, shown in Fig. 4, differed from previous RNA-seq studies by finding the transcript level of PDRP2 to be ~3-fold higher than that of PDRP1. When compared with the C_4_ PPDK transcript level, a representative high-copy mRNA in maize (Ponnala *et al.*, 2014; Stelpflug *et al.*, 2016), both PDRP transcripts are clearly in the lower abundance range (e.g. ~5–15% of the PPDK level). Transcript levels were not similarly determined for isolated MCs and BSCs out of concern for changes in mRNA that may take place during the 2–3 h isolation procedure of MC protoplasts (Covshoff *et al.*, 2013; Döring *et al.*, 2016).

### Immunoblot analysis of PDRP1 and PDRP2 in proteins extracted from isolated mesophyll protoplasts, bundle sheath strands, and intact leaves

**Fig. 5.**
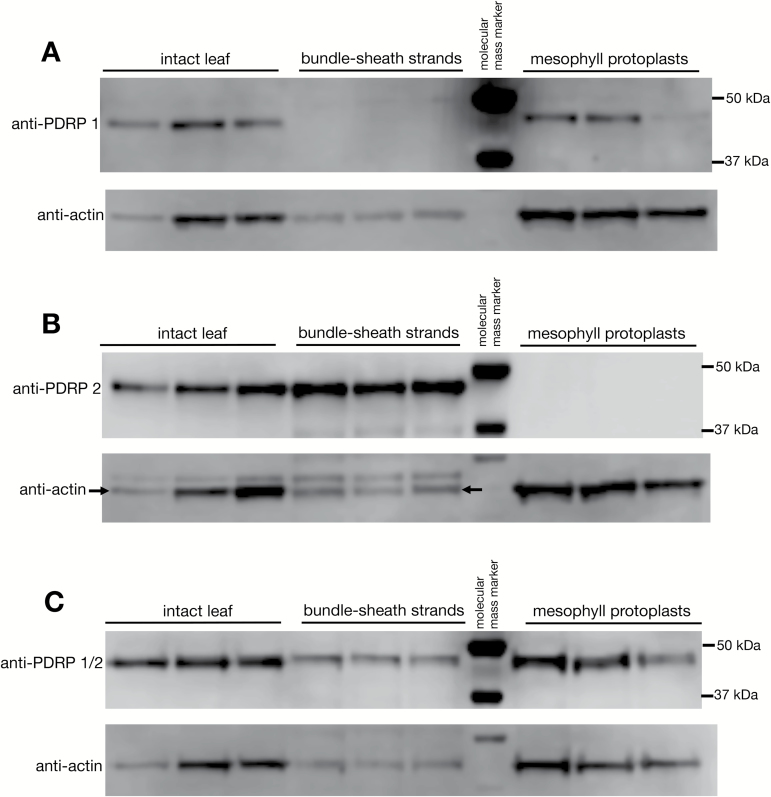
Immunoblot analysis of protein extracted from isolated BSC strands, MC protoplasts, and intact leaves. Blots were probed with affinity-purified peptide antibodies against PDRP1 (A), PDRP2 (B), and PDRP1/2 (C). Each lane represents protein extracted from independent isolations of BSC strands, MC protoplasts, and leaf material. Protein per lane: intact leaf=15 µg, BS=7.5 µg, M cell=13 µg. Following chemiluminescent detection, blots were stripped of primary antibody and reprobed with an anti-actin antibody as a lane load control.

Identical immunoblots of protein extracted from MC protoplasts, BSC strands, and intact leaves were probed separately with anti-PDRP1, anti-PDRP2, and anti-PDRP1/2 antibodies (Fig. 5). In the anti-PDRP1-probed immunoblot (Fig. 5A), a strong band corresponding to the predicted molecular mass of PDRP1 (~45 kDa) was detected in two out of the three MC replicates (and see Supplementary Fig. S3 for additional replicate lanes of MCs with anti-PDPR1). A PDRP1 band was detected in all replicates of intact leaf but was absent in all BSC replicate lanes. In the anti-PDRP2-probed immunoblot (Fig. 5B), a band corresponding to the predicted molecular mass of PDRP2 (~43 kDa) could be detected in BSC strand and intact leaf lanes but was absent in the MC lanes. In the anti-PDRP1/2 antibody-probed blot, PDRP could be detected in leaf, BSC strands, and MC lanes, confirming the presence of PDRP1 and PDRP2 on the blots shown in Fig. 5A and B. It should be noted that band signal strength of the respective anti-PDRP1- and anti-PDRP2-probed blots cannot be used as an indication of comparative abundance since slightly different epitopes were used to generate the respective polyclonal antibodies (Fig. 2). This would not be the case for the PDRP1/2-probed blots (Fig. 5C), where a common epitope was used to generate the antibody (Fig. 2), and the signal strength of the PDRP bands can be presumed to reflect the relative abundance of each polypeptide. Under this assumption, PDRP1 and PDRP2 appear to be comparable in terms of polypeptide abundance in their respective cell type relative to the actin signal. It should be further noted that with only a minor, ~2 kDa difference, the gel system used was insufficient to resolve distinct PDRP1 and PDRP2 bands in the PDRP1/2-probed blots.

### Recombinant PDRP1 and PDRP2 enzyme activity assays

**Fig. 7.**
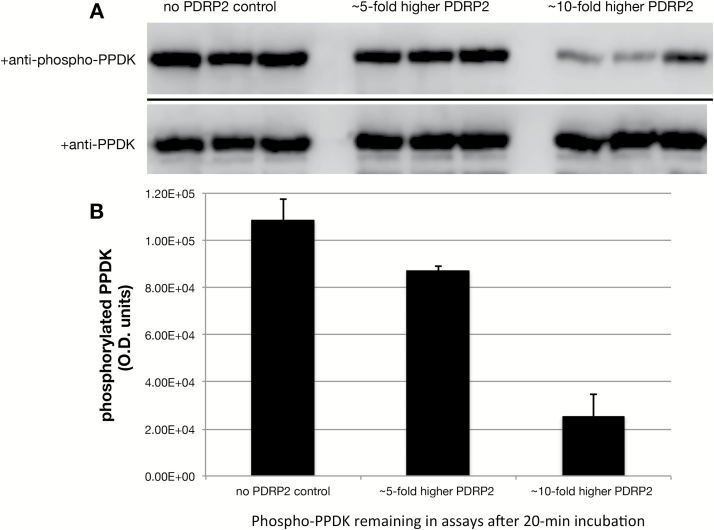
Effect of elevated levels of PDRP2 on *in vitro* phosphotransferase activity. End-point *in vitro* assays of PDRP2 PPT activity were carried out as in (Fig. 6B), except that PRDP2 in the assays was increased from 6 ng ml^−1^ to 30 ng ml^−1^ (5-fold) and 60 ng ml^−1^ (10-fold). (A) Immunoblots of PPT reaction aliquots probed with phospho-PPDK antibody. Phospho-PPDK bands in each lane were generated from separate PPT assays. Blots were stripped and reprobed with PPDK antibody as a loading control. (B) Mean signal intensities of the phospho-PPDK bands above were replotted in graphical form. (*n*=3; values are the means ±SE).

The bifunctional enzyme properties of PDRP1 have been fully elucidated, as reported in previous studies (Burnell and Hatch, 1983, 1985; Roeske and Chollet, 1987; Chastain, 2011, and references therein). We therefore utilized PDRP1 as a ‘control’ PDRP for assessing the enzyme functions of PDRP2 in side by side, *in vitro* PK and PPT assays. Using an immunobased protocol previously developed for assay of PDRP1 (Chastain *et al.*, 2011; Tolentino *et al.*, 2013), linear rates of PDRP-catalyzed PPDK phosphorylation (Fig. 6A) and PPDK-P dephosphorylation (Fig. 6B) were obtained. From these, specific enzyme activities for PK and PPT were calculated (Fig. 7C). For the PK enzyme function, we found both isoforms to be comparable in terms of specific activity [17.2 optical density units (ODU) μg^−1^ h^−1^ for PDRP2 versus 15.1 ODU μg^−1^ h^−1^ for PDRP1). For the PPT enzyme function, a specific activity of 7.0 ODU μg^−1^ h^−1^ for PDRP1 was obtained. The lower specific activity of PDRP1’s PPT versus PK is in agreement with previously reported specific activities for partially purified maize PDRP1 (Burnell and Hatch, 1985; Roeske and Chollet, 1987; Smith *et al.*, 1994). Notably, we were unable to detect PPT activity for the PDRP2 isoform, even when the assay time was extended from 12 min to 28 min (Fig. 6B). However, when the amount of PDRP2 enzyme used in the reaction was increased from 6 ng ml^−1^ (Fig. 6B) to 30 ng ml^−1^ (5-fold) and 60 ng ml^−1^ (10-fold), a detectable dephosphorylation of phospho-PPDK was observed at the end of the 20 min incubation period (Fig. 7A, B). The PPT specific activity estimated from these end-point assays was 0.5 and 1.1 ODU μg^−1^ h^−1^, respectively.

### Immunolocalization of PPDK and PDRP2 in maize leaves

**Fig. 8.**
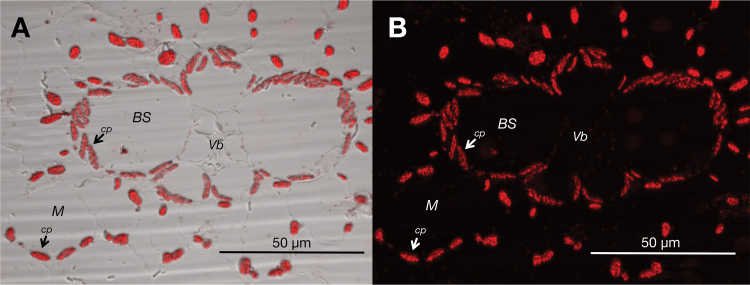
*In situ* immunolocalization of PPDK in chloroplasts of maize leaves via confocal laser microscopy. Shown are contrasting images of the same immunolabeled cross-section: (A) light-field contrast image revealing cell walls surrounding PPDK-containing MC and BSC chloroplasts; (B) dark-field contrast revealing the distinct outline of the bundle sheath as indicated by the stained PPDK in MC and BSC chloroplasts. Scale bar=50 µm; Vb, vascular bundle; cp, chloroplast. (This figure is available in colour at *JXB* online.)

**Fig. 9.**
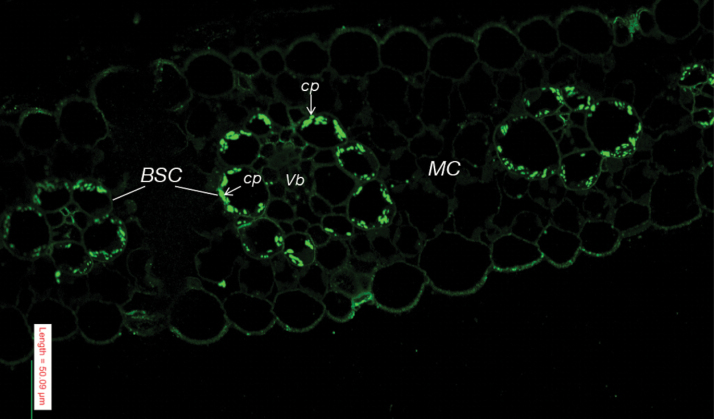
*In situ* immunolocalization of PDRP2 in BSC chloroplasts of maize leaves via confocal laser microscopy. Shown is a maize leaf cross-section immunolabeled with PDRP2 antibody and Alexa-488-labeled secondary antibody. Scale bar=50 µm; Vb, vascular bundle; cp, chloroplast. (This figure is available in colour at *JXB* online.)

Previous investigations that assessed how C_4_ enzymes are distributed intercellularly between isolated MCs and BSCs relied on enzyme assays of C_4_ enzymes in cell-specific extracts and/or immunoblots of cell-specific extracts probed with C_4_ enzyme-specific antibodies. Although these approaches have correctly defined the cell types for which the C_4_ enzymes are abundantly expressed, residual amounts of C_4_ enzyme can be expected to be present in extracts from the opposing BSC or MC type (Edwards *et al.*, 2001; Chang *et al.*, 2012; Döring *et al.*, 2016). This is usually attributed to imperfections inherent in the cell separation techniques so that some amount of cell type cross-contamination is inevitable. Thus, it is not entirely possible to know from these studies if the low amounts of C_4_ enzyme detected in the opposing C_4_ cell type is from cross-contamination or is endogenous in origin. However, in our investigation, a critical question was to establish whether PDRP2 and its regulatory target, C_4_ PPDK, were conclusively present in BSC chloroplasts. We therefore utilized immunolocalization for direct *in situ* imaging of immunolabeled PPDK and PDRP2 in fixed maize leaf cross-sections. As evident in Fig. 8, PPDK was detected in chloroplasts of MCs, as expected, but also significantly in chloroplasts of BSCs. We also demonstrate that PDRP2 is exclusively localized to BSC chloroplasts (Fig. 9), as had been predicted by the ChloroP algorithm (Emanuelsson *et al.*, 1999).

## Discussion

### PDRP2 as a possible regulator of PPDK in BSC chloroplasts

PDRP2 was first discovered following the release of version-2 of the maize B73 genome (Andorf *et al.*, 2016). It is highly similar (90%) to PDRP1 in terms of its primary structure (Fig. 2) and, as with PDRP1, is predicted to encode an N-terminal chloroplast transit peptide (Emanuelsson *et al.*, 1999). The discovery of the PDRP2 gene in maize was not unexpected, but the observation that its transcript was expressed in BSC strands mutually exclusive of the MC-specific PDRP1 transcript was. In the present study, we extend this observation by providing evidence to show that the expressed polypeptides of PDRP2 and PDRP1 are also BSC and MC specific, respectively. We also show that the transcript and polypeptide for PDRP2 are expressed at levels comparable with PDRP1. Furthermore, we provide evidence that PDRP2 and its putative regulatory target C_4_ PPDK, are co-localized in BSC chloroplasts. The substantial amount of PPDK that was observed in BSC chloroplasts was also somewhat unexpected. C_4_ pathway studies from the previous era viewed PPDK, in accordance with its role in the C_4_ pathway, as strictly a mesophyll-localized enzyme (Kanai and Edwards, 1999). However, for maize, a number of these studies had specifically observed a relatively higher level of PPDK in BSC chloroplasts (Aoyagi and Nakamoto, 1985; Sheen and Bogorad, 1987; Langdale *et al.*, 1988; Ueno, 1998). More recent investigations have found surprisingly high levels of C_4_ PPDK transcript in BSCs relative to MCs (Chang *et al.*, 2012; Tausta *et al.*, 2014). In this respect, C_4_ PPDK may be unique among the other differentially expressed C_4_ pathway enzymes by its relatively high expression in the opposing cell type. Thus, the significant amounts of C_4_ PPDK we observed in BSC chloroplasts may be an indication that differential expression of the C_4_ PPDK gene between MC and BSCs may not be as stringently regulated, either transcriptionally or post-transcriptionally, as it is for the other C_4_ pathway enzymes. In turn, such ‘leaky’ expression of the C_4_ PPDK transcript may have contributed to the selection of a BSC-specific PDRP isoform to regulate PPDK enzyme that accumulates in this compartment.

### *In vitro*, PDRP2 is essentially a monofunctional regulatory enzyme

In our assays of PDRP1 and PDRP2 enzyme activities, it was discovered that PDRP2 essentially lacks a PPT (PPDK-P dephosphorylation) activity while possessing essentially the same PK- (PPDK phosphorylation) specific activity as the bifunctional PDRP1 isoform. When the level of PDRP2 in the assay was elevated 10-fold, a PPT activity for PDRP2 could be detected. The *in vivo* relevance of this observation is unclear. The high level of PDRP that was used in the assays to detect PPT activity (i.e. 60 ng ml^−1^) may far exceed the endogenous concentration range of PDRP2 that exists in BSC chloroplasts, although this has yet to be determined. Recently, the crystal structure for maize PDRP1 has been elucidated, and structure–function relationships defined for many of the domains and residues (Jiang *et al.*, 2016). Using this detailed structural information, we searched for changes in critical residues in our alignment of PDRP2 relative to PDRP1 that could account for the changes we observed in PDRP2 enzyme function: the lack of PPT activity at lower PDRP concentrations and the marginal gain in PPT activity at extremely high concentrations. In our analysis, none of the PDRP1 residues shown by this previous study to be directly involved in catalysis was altered in PDRP2. However, we did find one key residue involved in PDRP1 dimerization (Cys417) that was replaced with a tyrosine in PDRP2 (Tyr411) (Fig. 2). Cys417 in the PDRP1 crystal structure was demonstrated to form an intermolecular disulfide bond with Cys128 from within an adjacent monomer. This bond forms one of four disulfide bridges within the dimeric enzyme (two intermolecular and two intramolecular) that stabilize the PDRP1 homodimer, the native conformation of the holoenzyme. Presumably, the inability of PDRP2 to form this intermolecular bond would impair dimer stability of the PDRP2 holoenzyme. How this could lead to the observed changes in PDRP2 enzyme function has yet to be explored, but suggests a mechanism involving monomer–dimer equilibrium, with monomeric PDRP2 catalyzing the PK reaction but not the PPT reaction (lower PDRP2 concentrations) and dimeric PDRP2 competent at catalyzing both reactions (higher PDRP2 concentration). Such a mechanism may also account for the very low PPT activity observed for the Arabidopsis PDRP isoform *At*RP2 (Chastain *et al.*, 2008, 2011; Astley *et al.*, 2011), as suggested by its alignment to the *Zm*PDRP1 primary sequence (Jiang *et al.*, 2016). Like *Zm*PDRP2, *At*RP2, and its fully bifunctional counterpart *At*RP1, are shown to lack the conserved cysteine residue at the Cys417 position in *Zm*PDRP1. However, *At*RP2, but not *At*PR1, also lacks an additional pair of conserved cysteines (Cys165 and Cys171 in *Zm*PDRP1) shown to form an intramolecular, dimer-stabilizing disulfide bridge in the *Zm*PDRP1 crystal structure (Jiang *et al.*, 2016). In these respects, *At*RP2 resembles *Zm*PDRP2 in its low PPT activity and in its deficiency of key disulfide bond-forming cysteine residues that promote dimer stability in *Zm*PDRP1. However, it has yet to be determined if the reduced PPT activity of *At*RP2 can be affected by increasing the enzyme concentration, as was observed for *Zm*PDRP2 in this study. Alternatively, the lack of a PPT activity we observed for *Zm*PDRP2 could be an artifact of recombinant expression of the enzyme in *E. coli*, such as improper folding. However, the finding that PDRP2’s *in vitro* PK-specific activity is comparable with that of the PDRP1 isoform would argue against such a recombinant-unique artifact as the PK function of the enzyme would likely be similarly impaired.

### A hypothesis for PDRP2 function in BS cells

In order to resolve the precise function of the PDRP2 isoform in maize leaves, it will be necessary to establish if, indeed, the enzyme lacks PPT activity *in vivo* and which is the focus of future experiments. Should this be the case, all the BSC chloroplast-localized PPDK would be expected to be phosphorylated and rendered inactive during each dark cycle via PDRP2’s robust PK activity. Alternatively, in the light, dephosphorylation of inactive PPDK-P would not occur due to PDRP2’s lack of PPT activity. Hence, any newly synthesized PPDK imported into BSC chloroplasts would accumulate as inactive enzyme. Notably, there is no evidence to date to suggest that any other enzyme (e.g. PP2A and PP2C protein phosphatases) besides PDRP can catalyze the dephosphorylation of PPDK-ThrP (JNB, unpublished). Under this scenario, we hypothesize that PDRP2 fulfills a useful role in C_4_ photosynthesis by suppressing C_4_ PPDK activity in BSC chloroplasts, assuming the PPDK in this compartment competes negatively for pyruvate with the PPDK activity in MC chloroplasts and the latter’s pivitol role in the C_4_ pathway.

## Supplementary data

Supplementary data are available at *JXB* online.

Fig. S1. Agarose gel analysis of total RNA isolated from maize leaves.

Fig. S2. Cross-contamination analysis of BSC strand and MC protoplast isolations.

Fig. S3. Additional replicates for anti-PDRP1- and anti-PDRP2-probed immunoblots.

Supplementary Figures 1-3
