# Supplementary material for: Maize leaf PPDK regulatory protein isoform-2 is specific to bundle sheath chloroplasts and paradoxically lacks a Pi-dependent PPDK activation activity
Source: J Exp Bot. 2017 Dec 21;69(5):1171–81. doi: 10.1093/jxb/erx471 (PMC6019023; doi:10.1093/jxb/erx471)
Supplement: Supplementary Figures 1-3 [file erx471_suppl_supplementary_figures_1-3.pdf]

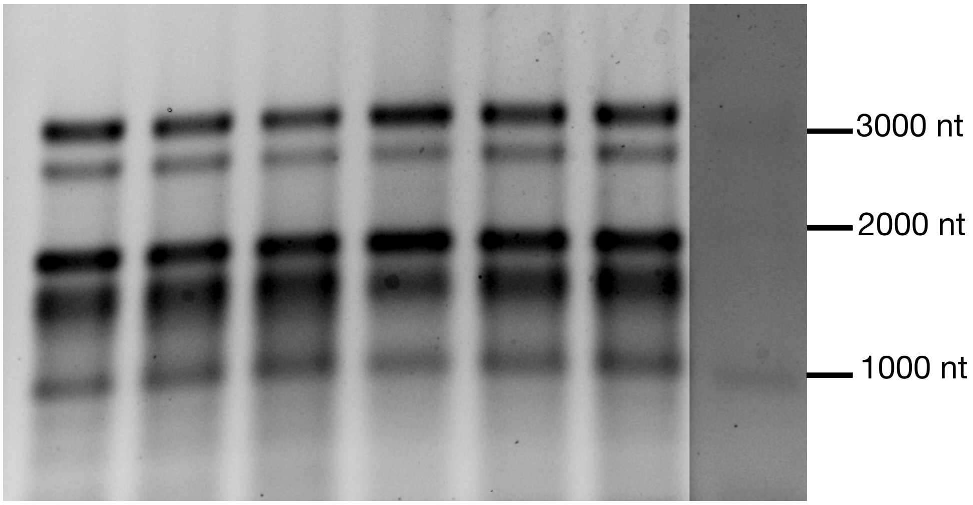

Fig. S1. Each lane is 5 µg of an independent isolation of maize leaf total RNA used for RTqPCR analysis.

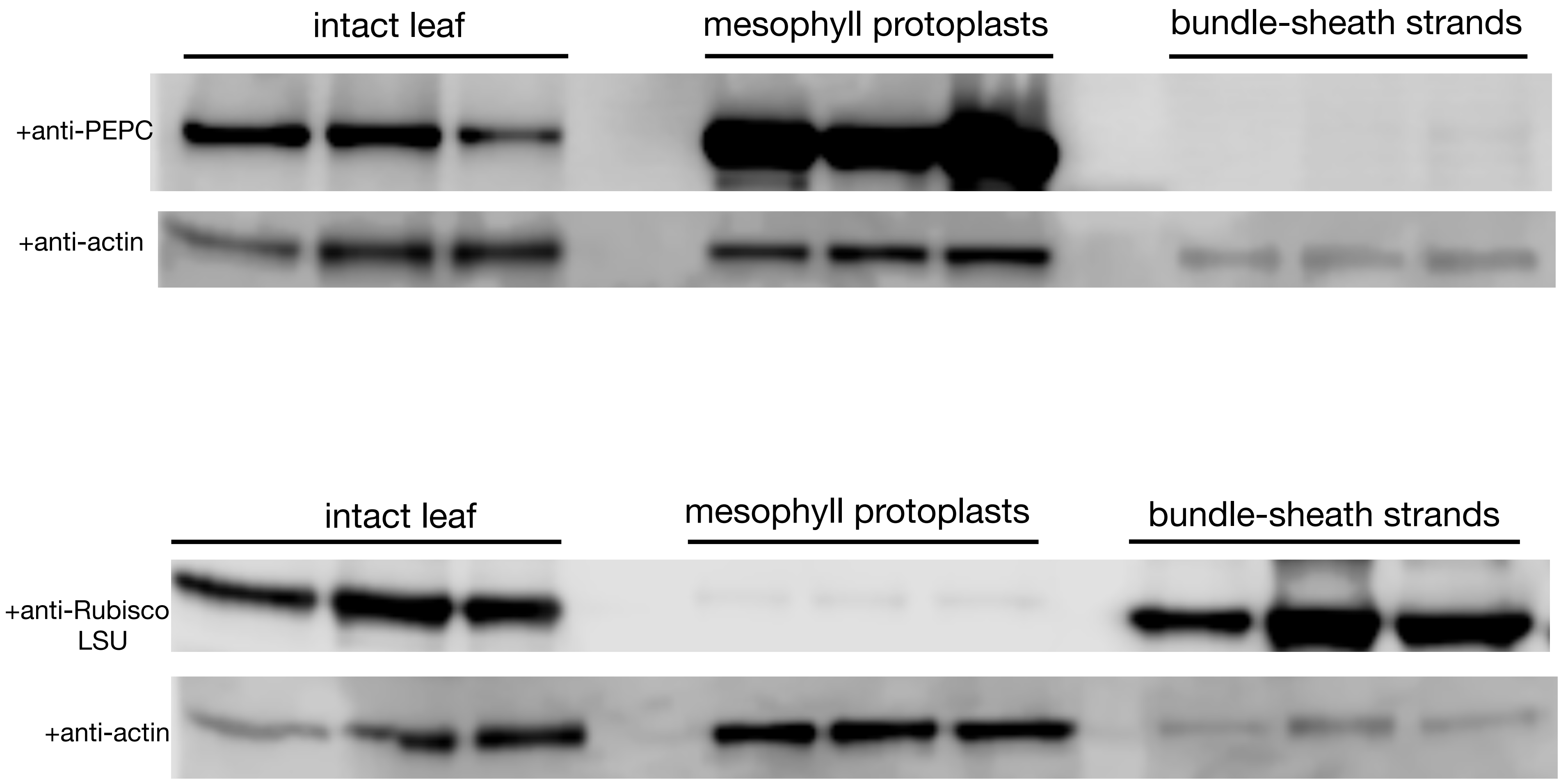

Fig. S2. Purity of isolated BS strand and M cell protoplast preparations was established by probing immunoblots of protein extracted from isolated BS strands, intact leaf, and isolated M protoplasts with antibodies to Rubisco LSU (as a BS-cell marker enzyme) and PEP carboxylase (M-cell marker enzyme). Each lane represents a separate BS or M-protoplast isolation with 4 μg protein per lane.

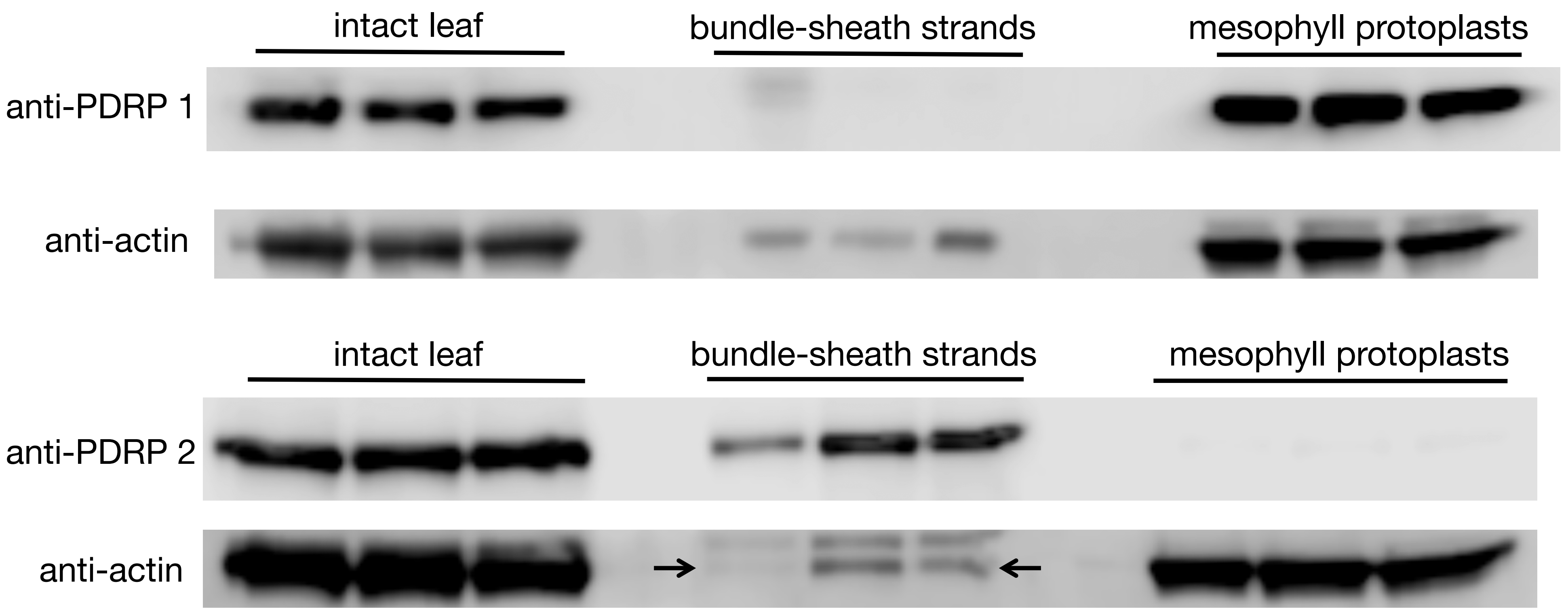

Fig. S3. Additional immunoblots augmenting the results shown in **Fig. 5**. Each lane represents protein extracted from an independent isolation separate from those used for the immunoblots shown in **Fig. 5**. Protein per lane: intact leaf = 17 μg, BS = 7.5 μg, MC = 14 μg.
